# Contributions of Artificial Intelligence to Analysis of Gut Microbiota in Autism Spectrum Disorder: A Systematic Review

**DOI:** 10.3390/children11080931

**Published:** 2024-07-31

**Authors:** Pau Climent-Pérez, Agustín Ernesto Martínez-González, Pedro Andreo-Martínez

**Affiliations:** 1 Department of Computing Technology, University of Alicante, 03690 San Vicente del Raspeig, Alicante, Spain; pau.climent@gcloud.ua.es; 2 Department of Developmental Psychology and Didactics, University of Alicante, 03690 San Vicente del Raspeig, Alicante, Spain; 3 Department of Agricultural Chemistry, Faculty of Chemistry, Regional Campus of International Excellence “Campus Mare Nostrum”, University of Murcia, Campus of Espinardo, 30100 Murcia, Spain; pam11@um.es

**Keywords:** artificial intelligence, autism spectrum disorders, gut microbiota, machine learning

## Abstract

Background: Autism spectrum disorder (ASD) is a highly heterogeneous neurodevelopmental disorder whose etiology is not known today, but everything indicates that it is multifactorial. For example, genetic and epigenetic factors seem to be involved in the etiology of ASD. In recent years, there has been an increase in studies on the implications of gut microbiota (GM) on the behavior of children with ASD given that dysbiosis in GM may trigger the onset, development and progression of ASD through the microbiota–gut–brain axis. At the same time, significant progress has occurred in the development of artificial intelligence (AI). Methods: The aim of the present study was to perform a systematic review of articles using AI to analyze GM in individuals with ASD. In line with the PRISMA model, 12 articles using AI to analyze GM in ASD were selected. Results: Outcomes reveal that the majority of relevant studies on this topic have been conducted in China (33.3%) and Italy (25%), followed by the Netherlands (16.6%), Mexico (16.6%) and South Korea (8.3%). Conclusions: The bacteria *Bifidobacterium* is the most relevant biomarker with regard to ASD. Although AI provides a very promising approach to data analysis, caution is needed to avoid the over-interpretation of preliminary findings. A first step must be taken to analyze GM in a representative general population and ASD samples in order to obtain a GM standard according to age, sex and country. Thus, more work is required to bridge the gap between AI in mental health research and clinical care in ASD.

## 1. Introduction

Autism spectrum disorder (ASD) is a neurodevelopmental disorder that is characterized by difficulties in social communication and interaction skills and the presence of restricted and repetitive patterns of behavior (RRBs) [1]. There has been an increase in the prevalence of ASD around the world [2,3,4,5]. This increase in prevalence may be influenced by different factors. One such factor is the validation of psychometric instruments, which has allowed progress in the detection and diagnosis of ASD, with the average age of global diagnosis being five years [6]. Furthermore, the recent development of cerebral organoids provides a powerful tool for studying both normal human embryonic brain development and, potentially, the origins of neurodevelopmental disorders, including ASD [7]. However, a series of epigenetic factors must also be considered that could influence the development of ASD [8]. In this sense, publications on gut microbiota (GM) and ASD have also seen a steady rise in the number [9,10].

Studies suggest that a series of symptoms exist that are associated with a possible dysbiosis in GM. Alterations to the gut microbiota–brain axis in ASD samples are often suspected following the appearance of pain and gastrointestinal symptoms (e.g., abdominal pain and constipation) [9,11,12]. Furthermore, sensory hyper-reactivity is highly related to selective or restrictive eating patterns (e.g., being a very picky eater [13,14]) and such types of restricted behavior can contribute to an increase in gastrointestinal symptoms [15] and poor intestinal functioning [9,11,12]. In fact, one meta-analysis concluded that individuals with ASD have dysbiosis in the genus *Bifidobacterium* [9]. In addition, such bacteria are beneficial for inhibiting the growth of pathogens and modulating the immune system and they can be used as a probiotic [10,16].

The composition of GM is highly diverse and varies widely between populations as a function of dietary, cultural and biological factors [17,18,19]. Analysis of GM is fundamentally performed through pyrosequencing of the 16S rRNA bacterial gene (e.g., V2–V3 regions) in fecal samples [13]. 16s rRNA sequencing is a culture-free method that is used to identify and compare bacterial diversity. Application of this analytical technique must follow a highly rigorous methodology in order to avoid sample contamination.

With regard to artificial intelligence (AI), it is important to, first, introduce a number of concepts that will be employed throughout the text and must be explained to practitioners of non-engineering-related fields (i.e., practitioners outside of computer science and similar fields). Artificial intelligence is a broad term in computer science that was introduced in the 1960s and relates to any capability of a computer to replicate human intelligence, reasoning, decision-making or, even, perception and cognition [20,21]. It entails aspects such as computer vision (CV) and pattern recognition (PR), amongst other aspects. Within AI, a field exists that is dedicated to ‘learning’. For example, machine learning (ML) is capable of discerning patterns that differ from other repeated patterns and, based on similarities between said patterns, classify traits described by features or vectors of features as pertaining to a class or ‘label’. For instance, merging the fields of CV and ML gives rise to object recognition, which aims to discern different object types or ‘labels’ based on features such as shape, texture and color. From this, an internal ‘model’ of the observed reality is created, which provides an internal representation that mirrors a mathematical function for distinguishing between the different types of patterns observed during the ‘training’ process [22,23,24].

Furthermore, artificial neural networks (ANNs) are a type of ML model inspired by the synapsis process of neurons in the visual cortex of biological models, although they are simplified mathematical versions and have, since their inception, varied substantially from their biological counterparts [25]. By arranging neurons that perform very simple mathematical operations (a product of ‘weight’ with the addition of a ‘bias’) in layers, akin to their arrangement in the visual cortex, they are capable of learning increasingly complex mathematical functions by combining outcomes produced at the level of previous layers. As will be seen, several of the reviewed works use this type of neural network for classification tasks as part of their analysis of gut microbiota composition. When ANNs are involved, and specifically if using models with more than one hidden layer (a type of layer that represents neither the input nor the output layer of neurons), a method termed ‘deep learning’ (DL) [26] comes into play. These types of methods have bloomed since the mid-2010s and constitute most methodologies employed in the present day for a wide range of applications. They are popular because of their good outcomes regarding computer vision, mostly in part to the emergence of convolutional layers and convolutional neural networks (CNNs) [27] but also in specialized models for natural language processing (NLP), amongst other fields. However, a caveat associated with their use pertains to the amount of data required for training such models, since the number of neurons (weights, biases) that constitute a neural network amount to tens or even hundreds of millions. Adjustment of the neuron’s internal representations during the decision-making process entails ‘seeing’ many potential scenarios during training and, therefore, makes the training process a computationally expensive endeavor.

Recently, computer-based AI has facilitated analysis, detection and diagnosis in mental health work. As AI techniques continue to be refined and improved, it will be possible to help mental health practitioners redefine ASD more objectively than is currently possible using the DSM-5, whilst also enabling earlier identification of ASD. Consequently, interventions will be able to be put in place earlier and will be more personalized [28]. Thus, the present study aims to conduct an updated systematic review of AI-generated findings regarding GM in ASD.

## 2. Materials and Methods

### 2.1. Protocol and Registration

The PRISMA checklist was followed when designing this systematic review [29]. The PRISMA approach ensures a systematic approach to research conducted in the field of ASD, GM and AI by framing critical analysis of key parameters, such as diagnosis via AI or ML.

The protocol used for this systematic review was not registered in any registry for systematic reviews or meta-analyses.

### 2.2. Eligibility Criteria

Eligibility criteria defining the scope of the present systematic review were agreed upon at a meeting of all authors and were applied to all papers retrieved from the database search. The following inclusion criteria were applied: (1) studies that examine diagnoses of ASD and GM made using AI or ML; (2) articles published prior to 25 April 2024 and (3) articles reporting comprehensive results and/or information. The following exclusion criteria were applied: (1) unsystematic narrative reviews; (2) studies published in a language other than English; (3) dissertations and conference proceedings; (4) books or book chapters; (5) editorial material; (6) articles examining ASD diagnosed according to any technique other than that already described as the main interest of the present study.

### 2.3. Information Sources

In order to minimize potential bias, literature searches were conducted in four different comprehensive databases. Namely, the comprehensive databases Web of Science (n = 23), Scopus (n = 41), PubMed (n = 22) and Science Database (n = 41) were searched. Works published prior to 25 April 2024 were included in these searches.

### 2.4. Search

The following search terms with relevant Boolean operators (including wildcards) were used: (gut* OR intestine* OR bowel* OR gastrointestinal*) AND (microbiota* OR microflora* OR bacteria* OR microbiome* OR flora* OR bacterial* OR bacteria* OR microorganism* OR feces* OR stool*) AND (‘Autism’ OR ‘ASD’ OR ‘autism spectrum disorder’) AND (‘Artificial Intelligence’ OR ‘Machine Learning’). Furthermore, Web of Science was searched according to ‘theme’, whilst Scopus was searched according to ‘title, abstract and keywords’, PubMed according to ‘all fields’ and finally, Science according to ‘all fields except full text (NOFT)’. No language restriction was applied to any of the searches.

### 2.5. Study Selection

Following the completion of database searches, a three-stage process was followed to review all records in accordance with the previously established eligibility criteria. First, titles were reviewed for eligibility, followed by abstracts and, finally, full texts. Articles gathered from all four databases were screened using ‘EndNote 20’ software with the aim of identifying duplicates and classifying papers according to inclusion/exclusion criteria. The three authors formed a ‘review team’, which took steps to minimize bias and possible random error at all stages of the review by independently verifying article selection according to title, abstract and full text. All three authors are experts in the fields of GM, AI and ASD.

### 2.6. Data Collection and Data Items

In order to ensure accuracy and impartiality at this step, data extraction was carried out by the review team. Using a data extraction form developed specifically for the present study, qualitative and quantitative data were extracted from the 12 articles included in this systematic review. The data items included for data extraction are given in Table 1.

## 3. Results

### 3.1. Study Selection

**Figure 1:**
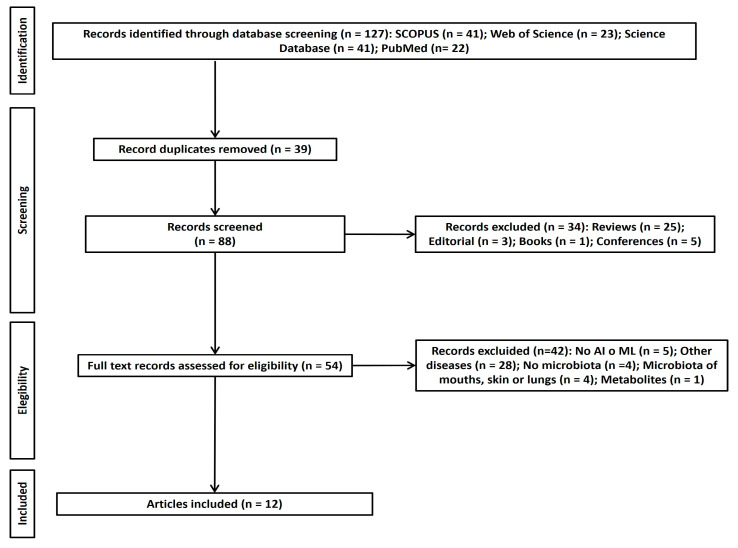
PRISMA flow chart of study selection.

As can be seen in Figure 1, 127 articles corresponding to the study aim or research question were selected. Following the elimination of duplicates using EndNote 20 software, the number of included studies was reduced to 88. A total of 34 articles were eliminated following the application of eligibility criteria. Despite five articles being deemed doubtful for final inclusion, 16 articles were included in the initial screening by the review team. After discussion between all authors, this number was reduced to 12 articles. Of the doubtful papers that were ultimately excluded, the paper of Shi et al. [62] was discarded due to its unsuitable format (letter to the editor), whilst of Liu et al. [63] was excluded for not focusing on ASD and being an unsuitable format (a proposal).

### 3.2. Individual Study Characteristics and Outcomes

The 12 included articles are summarized in Table 1 according to reference, participant characteristics, country, data source, study type, main findings, ML/DL method used and model used to predict microbiome.

The 12 included articles were published between 2020 and 2024 in five different countries (four [33.3%] in China, three [25.0%] in Italy, two [16.6%] in the Netherlands, two [16.6%] in Mexico and one [8.3%] in South Korea). Included articles were published in 10 different journals with 2 (16.6%) appearing in *Biomedicines*, 2 (16.6%) in *Frontiers in Microbiology* and 1 (8.3%) each in *Psychiatry Research*, *Neural Computing and Applications*, *Scientific Reports*, *BMC Bioinformatics*, *Computational and Structural Biotechnology Journal*, *Microorganisms*, *Microbial Pathogenesis* and *Microbiology Spectrum*.

Three (25%) articles gathered their own data to perform study analyses [30,45,46], whilst one (8.3%) article compared data they collected with data from two publicly available studies [36], one (8.3%) article exclusively used data from a publicly available study [34], three (8.3%) articles used data from two publicly available studies [31,47,50], one (8.3%) article used data from three publicly available studies [43], one (8.3%) article used data from five publicly available studies [52], one (8.3%) article used data from six publicly available studies [37] and one (8.3%) article used data from ten publicly available studies [56].

Some of the bacteria found to be implicated in ASD found by studies included in the present systematic review include *Bacteroides* [30,31,36,46], *Bifidobacterium* [36,43,46,56], *Prevotella* [36,46,50,52], *Faecalibacterium* [37,56], *Ruminococcus* [31,46,52] and *Clostridium* [31,36,37,43]. Outcomes indicate low levels of abundance pertaining to the bacteria *Bifidobacterium*, *Bacteroides*, *Prevotella*, *Ruminococcus*, *Lachnospira* and *Clostridium.* These findings suggest that these bacteria may be biomarkers of ASD.

### 3.3. Risk of Bias

The Methods Guide for Comparative Effectiveness Reviews was used to evaluate the risk of bias for each included article in the present systematic review [64]. This risk assessment method has also been used by the authors in other publications [13,65,66].

The strength of publications is expressed in terms of risk of bias: low (10–12); M = medium (6–9); H = high (1–5).

Five articles included in the present systematic review exhibited a low risk of bias, according to their risk of bias score, whilst seven studies exhibited a moderate risk of bias. Thus, a moderate risk of bias was found in 41.6% of included studies. In general, the greatest bias emerged in relation to sample size, identification of the sample and controlling for comorbidity factors such as intellectual disability, severity of ASD and diet. Overall and individual item scores for included articles are presented in Table 2.

Most studies examine samples from countries other than the original source of the publication. Data registries on GM come mainly from studies in China, Italy, the USA and Russia. Only four studies include an original sample [30,36,45,46], with such samples being highly limited and not representative of the ASD population [43,45,46].

### 3.4. Limitations

As previously discussed, the present systematic review was, by definition, limited by the databases used, the search terms chosen and the inclusion/exclusion criteria established [67].

## 4. Discussion

Present findings point to a relationship between low levels of abundance in the bacteria *Bifidobacterium*, *Bacteroides*, *Prevotella*, *Ruminococcus*, *Lachnospira* and *Clostridium* in ASD. This coincides with a previous meta-analysis study that found a dysbiosis or alteration of *Bifidobacterium* in ASD [9]. Thus, there outcomes produced using classic human methodology appear to coincide with those produced using ML. *Bifidobacterium* is possibly the bacterial biomarker most clearly implicated in the neurodevelopment of ASD. *Bifidobacterium* is one of the bacteria that appears most in studies with [30,36,43,45,46,56] and without AI in autism [9]. This is one of the first bacteria to colonize the intestine of neonates and is, therefore, highly important in neurodevelopment. Furthermore, it is associated with low levels of anxiety and gastrointestinal symptoms [10].

It can be observed that most of the machine learning and artificial intelligence (ML/AI) methodologies presented in the reviewed literature were employed for either (1) feature selection, i.e., to reduce the dimensionality of data and extract the most discriminative characteristics of data and, subsequently, use this subset of features for prediction, or as a support for the training of machine learning models; or (2) classification of ASD vs. HC (healthy controls), oftentimes also referred to as ‘logistic regression’, to be able to discern two types of gut bacteria profiles and, resultantly, determine the presence or absence of ASD-related dysbiosis in a new unseen sample.

One included study stands out [30] for performing regression for the prediction of microbial age instead of classification, whilst also notably employing extreme gradient boosting (XGB) to regress high/low SRS/VABS values.

However, a larger body of analyzed research uses very similar approaches, i.e., uses machine learning techniques, for sample classification as either ASD or HC participants. The techniques used vary but mostly correspond to ‘classical’ machine learning techniques, such as support vector machines (SVMs), whether the vanilla version [31,34,37] or kernelized with RBF (radial basis function kernel) [52], random forests (RF), k-nearest neighbors (k-NN) [34], gradient boosting (GB) [37,46], stochastic gradient descent (SGD) classifier [46], extra trees (ET) [46] and decision trees (DT) [52]. Random forest classifiers (RF) were the most commonly used method in the literature analyzed, as they tend to outperform other methods, and the complexity of training required is not very high. RF classifiers are employed in a number of included articles [31,34,37,46,47,50,56].

Another body of work focuses on the selection of features, with the two main aims of this being either direct biomarker discovery [36,43,45] or as a preliminary step towards simplifying model training within a machine learning classifier model [52,56] similar to those presented in the previous paragraph. For this, included studies present several methods, namely, principal component analysis (PCA), a classical method [45], recursive ensemble feature selection (REFS) [36,43], partial least squares discriminant analysis (PLS-DA) [45] and linear discriminant analysis (LDA) with effect size estimation (LEfSe) [52].

Finally, neural network-based models are seldom used in the studies analyzed. Indeed, only four following studies used classical models such as artificial neural networks (ANNs) [31,34] or multi-layer perceptron models (MLPs) [46,52]. From the descriptions available, it was not possible to determine whether ‘deep’ models were employed in these cases, although it seems implausible due to the nature of the methods used, which seem to refer to the prepackaged implementations available in off-the-shelf machine learning and data mining software libraries, or statistical analysis tools such as MATLAB, R, SPSS, Weka, Scikit-Learn, etc.

This leads to the first limitation inherent to included studies, which emerged as a lack of more advanced methods, although it was also the case that some deeper models would have encountered problems when dealing with low-dimensional data for which there are also too few samples. Data-driven learning, which is employed for deep neural network training, is ‘data-hungry’ in this sense and can lead to overfitting fairly easily. It would be interesting, however, to see more research conducted on this issue.

When using relatively small datasets, one of the most common techniques in ML/AI model training is to use techniques such as cross-validation (CV) or n-fold CV, which split data into one large and one small subset with the former being used for the main analysis and the latter (‘fold’) being used to determine model accuracy. Following the initial analysis, the second subset is then shifted, the system retrained, and average outcomes are calculated from the two resulting models. This has traditionally yielded good results; however, it does not provide additional information from other potentially existing broader datasets. For instance, it is now common in ML model training to perform transfer learning (TL) and, specifically, use fine-tuning processes during training. Within this training approach, the ML model is first trained on a larger domain (wider problem, e.g., general image classification) and then harnessed to address a restricted-domain problem (e.g., melanoma image classification). A similar approach, namely, domain adaptation (DA), could have been conducted here. When using DA, the aim is to learn from source data distribution, applying the model to different (yet related) target data distributions. This enriches the model, as it handles more data overall. In contrast, when using CV techniques, a very limited set of data is used; therefore, model overfitting becomes a common issue. In ASD GM studies, DA could be used to learn from pooled data from datasets originating from small-to-medium studies and applied to an unseen set of data from another small(er) study. Finally, it would also be interesting to introduce continual learning (CL) approaches, in which models are trained on ‘shifting’ datasets, i.e., data distributions that change over time, as more data are incorporated into the model, e.g., from growing corpora of collected ASD and HC GM-related data.

A second limitation of the studies included in the present review is that the majority are based on very small samples of 50 to 70 participants, which is far too low to support the conclusions drawn by the authors of these original studies. The risk of bias in each individual study must also be considered, not only because of the sample size, which is not very representative of the ASD population but also because of the poor representation of countries and cultures. Furthermore, it can be observed that the bacterial profiles used in the presented studies come from very specific populations and do not consider cultural differences in dietary intake, which is a key factor in the GM profiles of the general population in any given geographical area. It would be of interest for future work to conduct pooled analyses of these populations, whilst also completing the geographical profiles with additional data from less observed countries and regions. This could then be used to train a larger model, or even a deeper neural model, as the risk of bias and overfitting would be diminished. A more global examination of GM is necessary as a function of evolutionary periods within both neurotypical populations and those with ASD. In addition, ASD severity must be considered, alongside the existence of gastrointestinal symptoms. A huge challenge to research is posed by the standardization of global GM, which should also correspond to a preliminary step in the development of AI. At this time, more basic research is needed to analyze the diversity of GM in the general population.

In summary, the following strengths and weaknesses are seen in studies of AI in the study of GM in ASD: (1) acceptable samples for validation studies of psychometric instruments, which tend to imply few variables or factors with 250–500 individuals with ASD. However, variability in GM is greater because each family of bacteria acts as a higher dimension or factor that, in turn, includes thousands of genera and species of bacteria. Thus, samples should comprise at least 1000 individuals with ASD representing the three levels of autism severity according to the DSM-5. (2) ASD study samples should be representative of the rural and urban areas in the countries, in which the research is conducted and avoid extremes with regard to socioeconomic resources given that socioeconomic context influences diet [12,68] and autism severity [69]. (3) Additionally, GM studies should be performed with individuals with ASD who have comorbidities. (4) Due to the small study samples, correlations according to age and sex have not been performed with GM individuals with ASD. Future studies should consider these factors. (5) The use of AI should have a rigorous prior methodological basis based on sample size, age and evolutionary period. In this way, predictions about biomarkers could be more adjusted to the reality of GM in autism. In conclusion, more global and rigorous analyses regarding AI would be possible if all these methodological factors were considered. In any case, the incorporation of AI is an important advance that saves time and cuts economic costs in ASD research.

## 5. Conclusions

Current methods for the prediction of ASD in children based on GM data are still hampered by bias due to the small sample sizes used by relevant research. This limits the viability of such methods for use in a clinical setting, as they are still in need of further in-depth research. Further data are required to be able to construct large predictive models that can be generalized to global populations and consider diverse cultural and ethnic backgrounds. Generally speaking, larger samples are needed, comprising thousands of data points or more, in order to avoid the bias observed in the methods employed in the present review. Future studies should consider and strive to control for environmental factors such as diet and country of origin, as well as analytical factors such as sequencing platform and hypervariable region when comparing GM in patients with ASD with neurotypical patients. Finally, further research is required on this topic in order to facilitate early diagnosis and provide tools for a better prognosis of ASD in children and teenagers.

## Figures and Tables

**Table 1:** Characteristics of included studies.

Ref	Participant Characteristic		Country	Data Source	Study Type	Main Finding	ML/DL Method Used	Model Predicting Microbiome
	Experimental Group	Control Group						
[30]	ASD (n = 249)	NT siblings (n = 106) NT control (n = 101)	South Korea	Original	PCR, 16S rRNA (V3–V4 regions)	Negative association between *Bifidobacterium longum* and Childhood Autism Rating Scale outcomes, as well as a negative association between *Streptococcus salivarus* and Social Responsiveness Scale (SRS) outcomes in ASD.	ML: XGB regression of high/low SRS/VABS values Prediction of microbial age	*Bacteroides vulgatus*, *Roseburia cecicola group*, *Lachnospiraceae* and *Agathobaculum butyriproducience* showed significantly different abundances between high and low SRS groups in the E1 model (*p* ≤ 0.01), but not in the E2-SRS classification model. *Streptococcus salivarus* significantly differed between high and low SRS groups in model E2 (*p* ≤ 0.01), but not in E1.
[31]	ASD (n = 48) (from 2 to 7 years old, average 5, average BMI = 17.4, 10 females and 38 males) ASD (n = 77)	NT (n = 48) (all at 48 months, no allergies, 24 females and 24 males, average BMI = 16.3). NT (n = 50)	Mexico	[32,33]	16S rRNA (V3–V4 regions) (V4 region)	See [32,33]	ML: SVM, RF DL: ANNs Classification ASD v. HC	*Lachnospira* (primary predictor in the RF- and ANN-based models, ranking second in the SVM) Of the five main predictors in SVM and ANN models: *Bacteroides* (*p* = 2.4 × 10^−3^), *Escherichia–Shigella* (*p* = 2.39 × 10^−2^), *Akkermansia* (*p* = 2.51 × 10^−2^) and *Dialister* (*p* = 3.67 × 10^−2^) are statistically different. SVM [32]: *Bacteroides*, *Lachnospira*, *Blautia*, *Lachnoclostridium* and *Subdoligranulum* ANN [32]: *Lachnospira*, *Bacteroides*, *Lachnoclostridium*, *Blautia* and *Subdoligranulum* RF [32]: *Lachnospira*, *Escherichia–Shigella*, *Bacteroides*, *Blautia* and *Roseburia* ANN performed better than SVM on training and validation partitions, with 97.01% for training and 82.21% for validation. SVM [33]: *Ruminococcus torques*, *Anaerobutyricum* *Dorea*, *Subdoligranulum* and *Bacteroides* ANN [33]: *Anaerobutyricum*, *Bacteroides*, *Ruminococcus torques*, *Dorea* and *Subdoligranulum* RF [33]: *Anaerobutyricum*, *Faecalibacterium*, *Clostridium sensu stricto*, *Ruminococcus torques* and *Agathobacter*
[34]	ASD (n = 111)	NT (n = 143)	Mexico	[35]	16S rRNA (V4 region)	See [35]	ML: RF, SVM, kNN, NB DL: ANNs Classification ASD v. HC	Main predictor: *Prevotella_2.* Other significant predictors: *Ruminiclostridium_6* and the *Alloprevotella.* The ANN model demonstrates a 6% increase in sensitivity compared to kNN and RF models
[36]	ASD (n = 60) ASD (n = 77) ASD (n = 48)	Siblings (n = 57) HC (n = 50) HC (n = 48)	The Netherlands	Original [32,33]	16S rRNA (V3–V4 regions) (V4 region)	See [32,33]	ML: REFS Feature selection through REFS	ASVs: 26 ASVs for differential abundances. ↓*Actinobacteria phylum*, *Bifidobacterium and Collinsella* in ASD ↑*Bacteroidota phylum*, *Prevotellaceae* and *Parabacteroides* in ASD ↑bacterial taxa in ASD phenotype: *Clostridia*, *Sarcina* and *Parabacteroides*
[37]	ASD (n = 540)	HC (n = 419)	Italy	[35,38,39,40,41,42]	16S rRNA (Different regions)	See cited articles	ML: RF, SVM, GB Classification ASD v. HC	Main bacterial generates for all three algorithms: *Alloprevotella*, *Sutterella*, *Haemophilus*, Faecalibacterium and an unclassified *Clostridia ‘UCG 014’.* RF and the GBM algorithms: [*Eubacterium] siraeum_group*, *Tyzzerella*, *Negativibacillus*, *Muribaculaceae*, *Gastranaerophilales*, *Megamonas* and *Rombustia.* GBM and SVM algorithms: *Bacteroides* and *Subdoligranulum* identified as important. ↓*Alloprevotella* genus in ASD sample (abundance in ASD samples = 0.34 ± 0.20, abundance in HC samples = 0.12 ± 0.14). ↑*Parasutterella* (ASD samples = 0.57 ± 0.16, HC samples = 0.38 ± 0.17), *Haemophilus* (ASD samples = 0.57 ± 0.19, HC samples = 0.33 ± 0.17), *Faecalibacterium* (ASD samples = 0.86 ± 0.14, HC samples = 0.70 ± 0.21) and *Clostridiales UCG 14* (ASD samples = 0.60 ± 0.21, HC samples = 0.34 ± 0.17) in ASD.
[43]	ASD (n = 60) ASD (n = 77) ASD (n = 48)	HC (n = 57) HC (n = 50) HC (n = 48)	The Netherlands	[32,33,44]	16S rRNA (V3–V4 regions) (V4 region)	See cited articles	ML: REFS Feature selection: biomarker identification	Better performance in AUC and MCC compared to K-Best and 10-time random selection methods. ↓*Bifidobacterium*, *Enterobacteriaceae*, *Lachnospira*, *Lachnospiraceae* and *Clostridium* in ASD
[45]	ASD (n = 41)	NT (n = 35)	Italy	Original	PCR 16S rRNA (V3–V4 regions)	*Bifidobacterium* was negatively correlated with indole and skatole Positive correlations between *Carnobacteriaceae*, *Actinobacillus*, *Pepetostreptococcaceae*, pentanoic acid, 2.6-dimethyl-pyrazine, nonadecane and 3-methyl-butanoic acid.	ML: PCA, PLS-DA Feature selection: biomarker identification	Hist Gradient Boosting Classifier was the best performing model with 89% accuracy. VOCs associated with ASDs: methyl isobutyl ketone, benzeneacetaldehyde, phenyl ethyl alcohol, ethanol, butanoic acid, octadecane, acetic acid, skatole and tetradecanal (myristyl aldehyde) Positive correlations with OTUs-VOCs couples: *-Bifidobacteriaceae*/2-dodecanol -Serratia/benzyl alcohol *-Roseburia*/1-butanol *-Firmicutes*/butanoic acid *-Pasteurellaceae*/3-methyl 1-butanol.
[46]	ASD (n = 41)	NT (n = 35)	Italy	Original	PCR 16S rRNA (V3–V4 regions)	30 ASD with GI symptoms: 93% with a high level of severity Phylum level: ↓*Actinobacteria*, *Cyanobacteria* and TM7 in ASD ↑*Proteobacteria* and *Bacteroidetes* in ASD (*Bacteroidetes* was observed in the ASD without GI symptoms group) Family level: ↓*Coriobacteriaceae*, *Bifidobacteriaceae*, *Actynomicetaceae* and *(Tissierellaceae)* in ASD. ↑*Alcaligenaceae*, *Lactobacillaceae*, *Prevotellaceaeae* and *Bacteroidaceae* in ASD. ↑Bacteroidaceae and Lactobacillaceae ASD without GI symptoms. Genus level: ↑*Bacteroides* and *Klebsiella* in ASD. *Klebsiella* and *Lactobacillus* were higher in ASD without GI symptoms. ↓*Bifidobacterium* and *Actinomyces* in ASD. ASD-related microbial biomarkers (*p*-value < 0.05): -*Bacteroidetes/Proteobacteria*. *-Bacteroidaceae/Rikenellaceae/* *Lactobacillaceae/Prevotellaceae/Pasteurellaceae/Alcaligenaceae.* *-Bacteroides/Lactobacillus/ Prevotella/Klebsiella/Roseburia/Haemophilus/Sutterella.*	ML: LR, SGD, RF, ET, GB, XGB, etc. DL: MLP Classification ASD v. HC	Contextually, model classification analysis based on ML identified both KOs and ko pathways able to classify 73% of patients with ASD versus CTRLs (*p*-value < 0.05). Specific selected OTUs for ASD and CTRLs revealed the main bacteria: ↓*Bacteroides*, *Lactobacillus*, *Prevotella*, *Staphylococcus* and *Sutturella* in ASD ↓*Ruminococcus*, *Blautia*, *Coprococcus*, *Bifodobacterium* and *Streptococcus* in ASD.
[47]	ASD (n = 43)	TD (n = 31)	China	[48,49]	IgA detection via ELISA [49] StoolGen fecal DNA extraction kit (CWBiotech Co., Beijing, China) and NanoDrop 2000 (Thermo Scientific, Foster City, CA, USA). A total of 5 µg (or more) of DNA was required for library construction using the TruSeq DNA sample preparation kit (Illumina, San Diego, CA, USA) [48].	VFGM genes related to ASD: cpsH, cpsJ and cpsO genes related to high levels of *Streptococcus agalactiae 2603 V/R* in the gut of ASD children with/without GI symptoms	ML: RF Classification ASD v. HC	The main genes involved according to machine learning via the random forest method were mtrE, kfiC, pvdM and hasA.
[50]	ASD (n = 73)	TD (n = 71)	China	[48,51]	Illumina NovaSeq 6000 Illumina HiSeq 4000, Illumina Inc. San Diego, CA, USA	See cited articles	ML: RF Classification ASD v. HC	Predicted performance was evaluated according to AUROC. In the China cohort, a high AUROC value of 0.984 and 97% accuracy were achieved with only one round of a 100-iteration run. The Moscow cohort produced a poor average AUROC outcome of 0.81 and only 67% accuracy following six rounds of the 100-iteration run. Overall, average values for AUROC and accuracy were 0.86 and 80%, respectively, with an average feature set of 67 species. *Eubacterium_sp_CAG_248* and *Prevotella copri* were the most likely biomarkers involved in ASD.
[52]	ASD (n = 169)	NT (n = 128)	China	[39,42,53,54,55]	PCR 16S rRNA (Different regions)	See cited articles	ML: LDA (LEfSe)+ RF, kSVM + RBF, DT DL: MLP Feature selection + Classification	Dominant major genera: *↓Prevotella*, *↓Ruminococcus* and *Roseburia* as potential biomarkers of ASD. *Prevotella*, *Roseburia*, *Ruminococcus*, *Megasphaera* and *Catenibacterium* as potential biomarkers in ASD patients. However, only *Prevotella* significantly differed between the two groups.
[56]	ASD (n = 569)	HC (n = 450)	China	[32,33,35,38,40,41,46,57,58,59,60,61]	PCR 16S rRNA (V3–V4, V4, V4–V5 regions) Illumina MiSeq	See cited articles	ML: RF Classification of ASD v. HC (after feature selection)	AUC of the training set and verification set was 0.688 and 0.706. Dominant genera of the ASD group included *Lachnospiracea_incertae_sedis*, *Clostridium_XVIII*, *Eubacterium*, *Anaerostipes*, C*lostridium_sensu_stricto*, *Coprococcus*, *Dorea* and *Faecalibacterium*. Subgroup analysis followed different sequencing platforms to examine dominant genera in ASD. Dominant genera in the ASD group included *Eubacterium*, *Bifidobacterium*, *Blautia*, *Dialister*, *Coprococcus* and *Lachnospiracea_* *incertae_sedis.*

Note: ASD = autism spectrum disorder; NT = neurotypical; HC = healthy control; GS = gastrointestinal symptoms; w = with; w/o = without; AUC = area under the curve; AUROC = area under the receiver operating characteristic curve; VFGM = virulence factor-related gut microbiota; MCC = the Matthews correlation coefficient = MCC; ASVs = amplicon sequence variants; ML = machine learning; REFS = recursive ensemble feature selection; PCA = principal component analysis; PLS-DA = partial least squares-discriminant analysis; LDA = linear discriminant analysis; LEfSe = LDA effect size; SVM = support vector machines = SVM; RF = random forest classifier; kNN = k-nearest neighbor classifier; NB = naïve Bayes classifier; LR = logistic regression; SGD = Stochastic gradient descent classifier; ET = extra trees classifier; DT = decision trees classifier; GB = gradient boosting; XGB = extreme GB; kSVM + RBF = kernelized SVMs (RBF kernel); ANNs = artificial neural networks; MLP = multi-layer perceptron; DL = deep learning. Feature selection: REFS = recursive ensemble feature selection; PCA = principal component analysis; PLS-DA = partial least squares-discriminant analysis; LDA = linear discriminant analysis; LEfSe = LDA effect size. ML = machine learning: SVM = support vector machines; RF = random forest classifier; kNN = k-nearest neighbor classifier; NB = naïve Bayes classifier; LR = logistic regression; SGD = stochastic gradient descent classifier; ET = extra trees classifier; DT = decision trees classifier; GB = gradient boosting; XGB = extreme GB; kSVM + RBF = kernelized SVMs (RBF kernel). DL = deep learning: although very basic DL methodologies, the following have been identified: ANNs = artificial neural networks; MLP = multi-layer perceptron.

**Table 2:** Quality assessment outcomes for included studies.

Item	[31]	[34]	[36]	[37]	[43]	[45]	[46]	[47]	[50]	[52]	[56]	[30]
1. Clear stated aim	2	2	2	2	2	2	2	2	1	2	2	2
2. Appropriate study size	1	2	2	2	2	1	1	1	2	2	2	2
3. Identified and assessed	1	1	2	2	1	1	2	1	1	1	2	2
4. Blinding of participants and personnel	1	1	2	1	1	1	2	1	1	1	2	2
5. Other bias (controls for dietary intake, reports on comorbidity and ASD severity)	1	1	1	1	1	1	2	1	1	1	1	2
6. AI or ML in GM	2	2	2	2	2	2	2	1	1	2	2	2
TOTAL	8	9	11	10	9	8	11	8	6	9	11	12
Risk of bias	4	3	1	2	3	4	1	4	6	3	1	0
Risk of bias classification	M	M	L	L	M	M	L	M	M	M	L	L

Note: AI = artificial intelligence; ML = machine learning; GM = gut microbiota; 0 = not reported; 1 = not adequately assessed; 2 = adequately assessed; L = low (10–12); M = medium (6–9); H = high (1–5).

## Data Availability

Not applicable.
